# Supplementary material for: Rodent trapping studies as an overlooked information source for understanding endemic and novel zoonotic spillover
Source: PLoS Negl Trop Dis. 2023 Jan 23;17(1):e0010772. doi: 10.1371/journal.pntd.0010772 (PMC9894545; doi:10.1371/journal.pntd.0010772)
Supplement: S1 Table — (DOCX) [file pntd.0010772.s001.docx]

## Supplementary Table 1

Supplementary Table 1: Data extraction tool for studies meeting inclusion criteria

| Extraction tool | Variable | Description |
| --- | --- | --- |
| Study data |  |  |
|  | link | link to manuscript |
|  | year_publication | year of publication |
|  | title | title of manuscript |
|  | journal_name | journal |
|  | aim_1 | stated aim of study |
|  | aim_2 | stated aim of study |
|  | aim_3 | stated aim of study |
|  | first_author | first author of the study |
|  | reference_uid | DOI/ISSN/ISBN of the publication |
|  | unique_id | unique ID for the current study |
|  | metric | measurement of species presence abundance/presence |
|  | trap_types | type of rodent traps used |
|  | trapping_method | construction of the sampling grid |
|  | repeated_visit | whether there were multiple study visits to the same sites |
|  | geolocation_level | the level of geolocation reported |
|  | speciation | the level of speciation of trapped rodents |
|  | aim | aim of the study dichotomised to Ecology or Zoonotic risk |
|  | aim_detail | categorisation of study aims |
|  | species_accumulation | whether a species accumulation curve to describe trapping effort is reported |
|  | diversity_measurement | whether there is a measure of rodent species diversity reported |
|  | trapping_effort | whether pathogens are assayed |
|  | pathogen | completeness of reported trapping effort |
| Rodent data |  |  |
|  | unique_id | unique ID for the current study |
|  | year_trapping | year rodent trapping occurred (range) |
|  | month_trapping | months trapping occurred (range) |
|  | country | country trapping occurred within |
|  | region | region trapping occurred within |
|  | town_village | name of towns or villages trapping occurred within |
|  | latitude_DMS_N | latitude of trapping site in degrees minutes seconds (North) |
|  | longitude_DMS_W | longitude of trapping site in degrees minutes seconds (West) |
|  | latitude_D_N | latitude of trapping site in decimal degrees (North) |
|  | longitude_D_E | longitude of trapping site in decimal degrees (East) |
|  | UTM_coordinates | location of trapping site in UTM coordinates |
|  | habitat | habitat type of trapping site |
|  | intensity_use | the intensity of human disturbance in the trapping site |
|  | genus | reported genus of trapped rodent/small mammal species |
|  | species | reported species of trapped rodent/small mammal species |
|  | number | number of trapped individuals |
|  | trap_nights | number of trap nights reported |
|  | capture_rate | rate of capture if reported |
|  | trap_night_unit | the unit of trap night measurement |
|  | study_nights | the number of study nights completed at the trap site |
| Pathogen data |  |  |
|  | unique_id | unique ID for the current study |
|  | year_trapping | year rodent trapping occurred (range) |
|  | month | months trapping occurred (range) |
|  | country | country trapping occurred within |
|  | region | region trapping occurred within |
|  | town_village | name of towns or villages trapping occurred within |
|  | habitat | habitat type of trapping site |
|  | genus | reported genus of trapped rodent/small mammal species |
|  | species | reported species of trapped rodent/small mammal species |
|  | pathogen_x | pathogens tested for, 1-7 possible columns |
|  | latitude_DMS_N | latitude of trapping site in degrees minutes seconds (North) |
|  | longitude_DMS_W | longitude of trapping site in degrees minutes seconds (West) |
|  | latitude_D_N | latitude of trapping site in decimal degrees (North) |
|  | longitude_D_E | longitude of trapping site in decimal degrees (East) |
|  | UTM_coordinates | location of trapping site in UTM coordinates |
|  | path_x_tested | number of individuals assayed for the corresponding pathogen, 1-7 possible columns |
|  | pcr_x_positive | number of individuals PCR positive for the corresponding pathogen, 1-7 possible columns |
|  | ab_ag_x_positive | number of individuals with positive serological assays for the corresponding pathogen, 1-7 possible columns |
|  | culture_x_positive | number of individuals culture positive for the corresponding pathogen, 1-7 possible columns |
|  | histo_x_positive | number of individuals histologically/histopathologically positive for the corresponding pathogen, 1-7 possible columns |
