# Supplementary material for: Rodent trapping studies as an overlooked information source for understanding endemic and novel zoonotic spillover
Source: PLoS Negl Trop Dis. 2023 Jan 23;17(1):e0010772. doi: 10.1371/journal.pntd.0010772 (PMC9894545; doi:10.1371/journal.pntd.0010772)
Supplement: S2 Table — (DOCX) [file pntd.0010772.s002.docx]

## Supplementary Table 2

Supplementary Table 2: Included studies

| Year publication | Author | Title | Journal/Publication |
| --- | --- | --- | --- |
| 1974 | D. C. D. Happold | The small rodents of the forest-savanna-farmland association near Ibadan, Nigeria, with observations on reproduction biology | Revue de Zoologie et de Botanique Africaines |
| 1974 | Thomas Monath | Lassa virus isolation from Mastomys natalensis rodents during an epidemic in Sierra Leone | Science |
| 1975 | Herta Wulff | Recent isolations of Lassa virus from Nigerian rodents | Bulletin of the World Health Organisation |
| 1975 | Malcolm Coe | Mammalian ecological studies on Mount Nimba, Liberia. | Mammalia |
| 1977 | D. C. D. Happold | A population study on small rodents in the tropical rain forest of Nigeria | La Terre et la Vie |
| 1977 | Sonia Jeffrey | Rodent Ecology and Land Use in Western Ghana | Journal of Applied Ecology |
| 1979 | J P Dedet | Isolation of Leishmania major from Mastomys erythroleucus and Tatera gambiana in Senegal (West Africa). | Annals of Tropical Medicine and Parasitology |
| 1982 | A Diallo | Bacteriological survey of leptospirosis in Zaria, Nigeria. | Tropical and geographical medicine |
| 1983 | C Robbins | Mastomys (rodentia: muridae) species distinguished by hemoglobin pattern differences. | The American journal of tropical medicine and hygiene |
| 1987 | Joseph McCormick | A prospective study of the epidemiology and ecology of Lassa fever. | Journal of Infectious Diseases |
| 1988 | J. Iyawe | Distribution of small rodents and shrews in a lowland rain forest zone of Nigeria, with observations on their reproductive biology | African Journal of Ecology |
| 1991 | J Trape | Tick-borne Borreliosis in West Africa | The Lancet |
| 1992 | Brian Mahy | Maintenance support of a field station in Sierra Leone, West Africa | US Army Medical Research and Development Command |
| 1993 | Laurent Granjon | Social structure in synanthropic populations of a murid rodent Mastomys natalensis in Senegal | Acta Theriologica |
| 1994 | Bruno Godeluck | A longitudinal survey of Borrelia crocidurae prevalence in rodents and insectivores in Senegal. | American Journal of Tropical Medicine and Hygiene |
| 1994 | G Diatta | A comparative study of three methods of detection of Borrelia crocidurae in wild rodents in Senegal | Transactions of the Royal Society of Tropical Medicine and Hygiene |
| 1995 | E Ikeh | Mastomys natalensis and Tatera gambiana as probable reservoirs of human cutaneous leishmaniasis in Nigeria. | Transactions of the Royal Society of Tropical Medicine and Hygiene |
| 1997 | C Mafiana | Gastrointestinal helminth parasites of the black rat (Rattus rattus) in Abeokuta, southwest Nigeria. | Journal of Helminthology |
| 1999 | Holger Meinig | Notes on the mammal fauna of the southern part of the Republic of Mali, West Africa. | Bonn Zoological Bulletin |
| 1999 | Jan Decher | Diversity and structure of terrestrial small mammal communities in different vegetation types on the Accra Plains of Ghana | Journal of Zoology |
| 2000 | Adrian Barnett | Ecology of rodent communities in agricultural habitats in eastern Sierra Leone: Cocoa groves as forest refugia | Tropical Ecology |
| 2000 | J Duplantier | Rodents as reservoir hosts in the transmission of Schistosoma mansoni in Richard-Toll, Senegal, West Africa | Journal of Helminthology |
| 2000 | James Ryan | Mammal fauna of the Muni-Pomadze Ramsar site, Ghana. | Biodiversity and Conservation |
| 2001 | Austin Demby | Lassa Fever in Guinea: {II}. Distribution and Prevalence of Lassa Virus Infection in Small Mammals | Vector-Borne and Zoonotic Diseases |
| 2001 | Khalilou Ba | Preliminary study on some rodents of southern Mauritania as reservoir of human pathogenic viruses | African Small Mammals |
| 2002 | Gauthier Dobigny | A cytotaxonomic survey of Rodents from Niger: implications for systematics, biodiversity and biogeography | Mammalia |
| 2002 | Laurent Granjon | The small mammal community of a coastal site of south-west Mauritania | African Journal of Ecology |
| 2003 | D Attuquayefio | A study of bushfires in a Ghanaian coastal wetland. Impact on small mammals | African Journal of Applied Ecology |
| 2003 | Laurent Granjon | The importance of cytotaxonomy in understanding the biogeography of African rodents: Lake Chad murids as an example | Mammal Review |
| 2004 | B. Sicard | Effects of climate and local aridity on the latitudinal and habitat distribution of Arvicanthis niloticus and Arvicanthis ansorgei (Rodentia, Murinae) in Mali | Journal of Biogeography |
| 2004 | Jan Decher | A rapid survey of terrestrial small mammals (shrews and rodents) of the Foret Classee du Pic de Fon, Guinea. | Rapid Assessment Program |
| 2004 | Sara Churchfield | First results on the feeding ecology of sympatric shrews (Insectivora: Soricidae) in the Tai National Park, Ivory Coast | Acta Theriologica |
| 2005 | Ara Monadjem |  | Conservation International |
| 2005 | D Attuquayefio | Preliminary biodiversity assessment (herpetofauna and mammals) of a coastal wetland in the Volta region, Ghana | Ghana Journal of Science |
| 2005 | Francesco Angelici | Patterns of specific diversity and population size in small mammals from arboreal and ground-dwelling guilds of a forest area in southern Nigeria | Journal of Zoology |
| 2005 | Jan Decher | Rapid assessment of Small Mammals at Draw River, {BoiTano}, and Krokosua Hills | Conservation International |
| 2005 | Laurent Granjon | Population dynamics of the multimammate rat Mastomys huberti in an annually flooded agricultural region of central Mali | Journal of Mammology |
| 2006 | Natalie Weber | A Rapid Survey of Small Mammals from the Atewa Range Forest Reserve, Eastern Region, Ghana | Conservation International |
| 2006 | Patrick Barriere | Rapid Survey of the Small Mammals of Ajenjua Bepo and Mamang River Forest Reserves, Ghana | Conservation International |
| 2006 | Ryan Norris | A rapid biological assessment of three classified forests in southeastern Guinea | RAP Bulletin of Biological Assessment 40 |
| 2007 | Joseph Fair | Lassa Virus-Infected Rodents in Refugee Camps in Guinea: A Looming Threat to Public Health in a Politically Unstable Region | Vector-Borne and Zoonotic Diseases |
| 2008 | Ayodeji Olayemi | Diversity and distribution of murid rodent populations between forest and derived savanna sites within south western Nigeria. | Biodiversity and Conservation |
| 2008 | D Attuquayefio | Biodiversity assessment (rodents and avifauna) of five forest reserves in the Brong-Ahafo Region, Ghana | Ghana Journal of Science |
| 2008 | G Raczniak | Cutaneous leishmaniasis in the Volta district of Ghana: An uncertain reservoir for focal disease outbreak | The Libyan Journal of Infectious Diseases |
| 2008 | Laurent Crespin | Annual flooding, survival and recruitment in a rodent population from the Niger River plain in Mali | Journal of Tropical Ecology |
| 2009 | Christiane Denys | New data on the taxonomy and distribution of Rodentia (Mammalia) from the western and coastal regions of Guinea West Africa | Italian Journal of Zoology |
| 2009 | Elisabeth Fichet-Calvet | Diversity and dynamics in a community of small mammals in coastal Guinea, West Africa | Belgian Journal of Zoology |
| 2009 | Ivoke Njoku | Studies on the seasonal variations and prevalence of helminth fauna of the black rat, Rattus rattus (L) (Rodentia: Muridae) from different microhabitats in Nsukka, Nigeria. | Animal Research International |
| 2010 | Adam Konecny | Indications of higher diversity and abundance of small rodents in human-influenced Sudanian savannah than in the Niokolo Koba National Park (Senegal). | African Journal of Ecology |
| 2010 | David Safronetz | Detection of Lassa Virus, Mali | Emerging Infectious Diseases |
| 2010 | Edward Omudu | A survey of rats trapped in residential apartments and their ectoparasites in Makurdi, Nigeria. | Research Journal of Agriculture and Biological Sciences |
| 2010 | Elisabeth Fichet-Calvet | Diversity, dynamics and reproduction in a community of small mammals in Upper Guinea, with emphasis on pygmy mice ecology | African Journal of Ecology |
| 2010 | Gauthier Dobigny | Molecular survey of rodent-borne Trypanosoma in Niger with special emphasis on T. lewisi imported by invasive black rats | Acta Tropica |
| 2010 | Jan Decher | Small mammal survey in the upper Seli River valley, Sierra Leone | Mammalia |
| 2010 | Lies Durnez | Terrestrial Small Mammals as Reservoirs of Mycobacterium ulcerans in Benin | Applied and Environmental Microbiology |
| 2010 | Mary Reynolds | A Silent Enzootic of an Orthopoxvirus in Ghana, West Africa: Evidence for Multi-Species Involvement in the Absence of Widespread Human Disease | American Journal of Tropical Medicine and Hygiene |
| 2010 | R Sall-Drame | Variation in cestode assemblages of Mastomys and Arvicanthis species (Rodents: Muridae) from Lake Retba in Western Senegal. | Journal of Parasitology |
| 2010 | Violaine Nicolas | Terrestrial small mammal diversity and abundance in central Benin: comparison between habitats, with conservation implications | African Journal of Ecology |
| 2011 | David Coulibaly-N'golo | Novel Arenavirus Sequences in Hylomyscus sp. and Mus (Nannomys) setulosus from Cote d'Ivoire: Implications for Evolution of Arenaviruses in Africa | PLOS One |
| 2011 | Karmidine Hima | Extensive Robertsonian polymorphism in the African rodent Gerbillus nigeriae: geographic aspects and meiotic data | Journal of Zoology |
| 2011 | Laurent Granjon | Guinean biodiversity at the edge: Rodents in forest patches of southern Mali | Mammalian Biology |
| 2011 | M. Thiam | Capacity for water conservation in invasive (Gerbillus nigeriae) and declining rodents (Taterillus pygargus and Taterillus gracilis) that exhibit climate-induced distribution changes in Senegal | Journal of Arid Environments |
| 2012 | Amawulu Ebenezer | Effects of Urbanization and Agricultural Expansion on the Upsurge of Wild Rats (Rattus rattus) in Yenagoa Metropolis of Bayelsa State, Nigeria | Research Journal of Applied Sciences, Engineering and Technology |
| 2012 | Christiane Denys | On a new species of Dendromus (Rodentia, Nesomyidae) from Mount Nimba, Guinea. | Mammalia |
| 2012 | Khalilou Ba | Ecology of a typical West African Sudanian savannah rodent community. | African Journal of Ecology |
| 2012 | Laurent Crespin | Demographic aspects of the island syndrome in two Afrotropical Mastomys rodent species | Acta Oecologica |
| 2012 | Tom Schwan | Endemic Foci of the Tick-Borne Relapsing Fever Spirochete Borrelia crocidurae in Mali, West Africa, and the Potential for Human Infection | PLOS NTD |
| 2013 | Adam Konecny | Invasion genetics of the introduced black rat (Rattus rattus) in Senegal, West Africa. | Molecular Ecology |
| 2013 | Blaise Kadjo | Assessment of terrestrial small mammals and a record of the critically endangered shrew Crocidura wimmeri in Banco National Park (Cote d'Ivoire) | Mammalia |
| 2013 | Gualbert Houemenou | Leptospira spp. Prevalence in Small Mammal Populations in Cotonou, Benin | ISRN Epidemiology |
| 2013 | Jean-Francois Trape | The epidemiology and geographic distribution of relapsing fever borreliosis in West and North Africa, with a review of the Ornithodoros erraticus complex (Acari: Ixodida). | PLOS One |
| 2013 | Joshua Kamani | Prevalence and diversity of Bartonella species in commensal rodents and ectoparasites from Nigeria, West Africa. | PLOS NTD |
| 2013 | Karl Kronmann | Two Novel Arenaviruses Detected in Pygmy Mice, Ghana | Emerging Infectious Diseases |
| 2013 | Reuben Garshong | Effect of Habitat Change through Infrastructural Development on Small Mammal Diversity and Abundance on the Legon Campus of the University of Ghana. | West African Journal of Applied Ecology |
| 2014 | Benjamin Ofori | Preliminary checklist and aspects of the ecology of small mammals at the University of Ghana Botanical Garden, Accra Plains, Ghana | Journal of Biodiversity and Environmental Sciences |
| 2014 | Elisabeth Fichet-Calvet | Lassa Serology in Natural Populations of Rodents and Horizontal Transmission | Vector-Borne and Zoonotic Diseases |
| 2014 | Madougou Garba | Spatial Segregation between Invasive and Native Commensal Rodents in an Urban Environment: A Case Study in Niamey, Niger | PLOS One |
| 2015 | Charles Narh | Source Tracking Mycobacterium ulcerans Infections in the Ashanti Region, Ghana | PLOS NTD |
| 2015 | Christelle Dassi | Detection of Mycobacterium ulcerans in Mastomys natalensis and Potential Transmission in Buruli ulcer Endemic Areas in CÃ´te d'Ivoire | Mycobacterial Diseases |
| 2015 | Georges Diatta | Borrelia infection in small mammals in West Africa and its relationship with tick occurrence inside burrows | Acta Tropica |
| 2015 | Pilar Foronda | Serological survey of antibodies to Toxoplasma gondii and Coxiella burnetii in rodents in north-western African islands (Canary Islands and Cape Verde). | Onderstepoort Journal of Veterinary Research |
| 2015 | R Mol | Small terrestrial mammal and amphibian survey BoÃ© region, Guinea-Bissau. | Silvavir Forest Consultants |
| 2015 | Thomasz Leski | Sequence variability and geographic distribution of Lassa virus, Sierra Leone. | Emerging Infectious Diseases |
| 2016 | Ayodeji Olayemi | Arenavirus Diversity and Phylogeography of Mastomys natalensis Rodents, Nigeria | Emerging Infectious Diseases |
| 2016 | Ayodeji Olayemi | New Hosts of The Lassa Virus | Scientific Reports |
| 2016 | Benjamin Ofori | Spatio-temporal variation in small mammal species richness, relative abundance and body mass reveal changes in a coastal wetland ecosystem in Ghana | Environmental Monitoring and Assessment |
| 2017 | Alexis Ribas | Whipworm diversity in West African rodents: a molecular approach and the description of Trichuris duplantieri n. sp (Nematoda: Trichuridae) | Parasitological Research |
| 2017 | C Lippens | Genetic structure and invasion history of the house mouse (Mus musculus domesticus) in Senegal, West Africa: a legacy of colonial and contemporary times | Heredity |
| 2017 | Christophe Diagne | Ecological and sanitary impacts of bacterial communities associated to biological invasions in African commensal rodent communities | Scientific Reports |
| 2017 | Christophe Diagne | Serological Survey of Zoonotic Viruses in Invasive and Native Commensal Rodents in Senegal, West Africa | Vector-Borne and Zoonotic Diseases |
| 2017 | Daniel Attuquayefio | Impact of mining and forest regeneration on small mammal biodiversity in the Western Region of Ghana | Environmental Monitoring and Assessment |
| 2018 | Ayodeji Olayemi | Widespread arenavirus occurrence and seroprevalence in small mammals, Nigeria | Parasites and Vectors |
| 2018 | Benjamin Ofori | Urban green area provides refuge for native small mammal biodiversity in a rapidly expanding city in Ghana | Environmental Monitoring and Assessment |
| 2018 | Carine Brouat | Seroprevalence of Toxoplasma gondii in commensal rodents sampled across Senegal, West Africa. | Parasite |
| 2018 | Isaac Clement | Endoparasites of Small Mammals in Edo State, Nigeria: Public Health Implications | The Korean Journal of Parasitology |
| 2018 | Joshua Kamani | Prevalence of Hepatozoon and Sarcocystis spp. in rodents and their ectoparasites in Nigeria | Acta Tropica |
| 2018 | Katharina Schaufler | Clinically Relevant {ESBL}-Producing K. pneumoniae {ST}307 and E. coli {ST}38 in an Urban West African Rat Population | Frontiers in Microbiology |
| 2018 | Kouame Akpatou | Terrestrial small mammal diversity and abundance in TaÃ¯ National Park, CÃ´te d'Ivoire | Nature Conservation Research |
| 2018 | Lokman Galal | Diversity of Toxoplasma gondii strains shaped by commensal communities of small mammals. | International Journal for Parasitology |
| 2018 | Marien Joachim | Movement Patterns of Small Rodents in Lassa Fever-Endemic Villages in Guinea | EcoHealth |
| 2018 | Stefano Catalano | Rodents of Senegal and their role as intermediate hosts of Hydatigera spp. (Cestoda: Taeniidae). | Parasitology |
| 2019 | Agnes Yadouleton | Lassa Virus in Pygmy Mice, Benin, 2016â€“2017 | Emerging Infectious Diseases |
| 2019 | Gualbert Houemenou | Pathogenic Leptospira in Commensal Small Mammals from the Extensively Urbanized Coastal Benin | Urban Science |
| 2019 | Joachim Marien | Evaluation of rodent control to fight Lassa fever based on field data and mathematical modelling. | Emerging Microbes and Infections |
| 2019 | Karmidine Hima | Native and Invasive Small Mammals in Urban Habitats along the Commercial Axis Connecting Benin and Niger, West Africa | Diversity |
| 2019 | Karmidine Hima | Population Dynamics and Genetics of Gerbillus nigeriae in Central Sahel: Implications for Rodent Pest Control | Ecology and Evolutionary Biology |
| 2019 | Kouame Akpatou | Assessment of Terrestrial Small Mammals in an Agro-industrial Company Concession, Western Liberia | International Journal of Applied Sciences and Biotechnology |
| 2019 | L Karan | Lassa Virus in the Host Rodent Mastomys Natalensis within Urban Areas of Nâ€™zerekore, Guinea | bioRxiv |
| 2019 | Moussa Diagne | Usutu Virus Isolated from Rodents in Senegal | Viruses |
| 2019 | Natalie Weber | New records of bats and terrestrial small mammals from the Seli River in Sierra Leone before the construction of a hydroelectric dam. | Biodiversity Data Journal |
| 2019 | Safianu Rabiu | Demographic response of the Gambian Gerbil to seasonal changes in Savannah fallow fields | Folio Oecologica |
| 2019 | Stefano Catalano | Plagiorchis sp. in small mammals of Senegal and the potential emergence of a zoonotic trematodiasis | IJP: Parasites and Wildlife |
| 2020 | Adama Diarra | Molecular Detection of Microorganisms Associated with Small Mammals and Their Ectoparasites in Mail | American Journal of Tropical Medicine and Hygiene |
| 2020 | Adama Zida | Mastomys natalensis, Cricetomys gambianus and Taterillus sp. were found {PCR} positive for Leishmania major in Burkina Faso, West Africa. | Annals of Parasitology |
| 2020 | Christophe Diagne | Association between temporal patterns in helminth assemblages and successful range expansion of exotic Mus musculus domesticus in Senegal | Biological Invasions |
| 2020 | Claire Stragier | Interplay between historical and current features of the cityscape in shaping the genetic structure of the house mouse ( Mus musculus domesticus) in Dakar (Senegal, West Africa) | Peer Community in Ecology |
| 2020 | Handi Dahmana | Rodents as Hosts of Pathogens and Related Zoonotic Disease Risk | Pathogens |
| 2020 | Henri-Joel Dossou | Invasive rodents and damages to food stocks: a study in the Autonomous Harbor of Cotonou, Benin. | Biotechnologie Agronomie Societe et Environnement |
| 2020 | Joachim Marien | Households as hotspots of Lassa fever? Assessing the spatial distribution of Lassa virus-infected rodents in rural villages of Guinea | Emerging Microbes and Infections |
| 2020 | Laurent Ahissa | Species composition and community structure of terrestrial small mammals in TanoÃ©-Ehy Swamp Forest (South-East Ivory Coast): implication for conservation | Nature Conservation Research |
| 2020 | Leonce Kouadio | Detection of possible spillover of a novel hantavirus in a Natal mastomys from Guinea. | Virus genes |
| 2020 | Stefano Catalano | Multihost Transmission of Schistosoma mansoni in Senegal, 2015-2018 | Emerging Infectious Diseases |
| 2020 | Violaine Nicolas | Small mammal inventory in the Lama forest reserve (south Benin), with new cytogenetical data | Journal of Vertebrate Biology |
| 2021 | Chibuisi Alimba | Wild black rats (Rattus rattus Linnaeus, 1758) as zoomonitor of genotoxicity and systemic toxicity induced by hazardous emissions from Abule Egba unsanitary landfill, Lagos, Nigeria. | Environmental science and pollution research international |
| 2021 | El Hadji Ndiaye | Tick-borne relapsing fever Borreliosis, a major public health problem overlooked in Senegal | PLOS NTD |
| 2021 | Mnqobi Mamba | Small mammals of a West African hotspot, the Ziama-Wonegizi-Wologizi transfrontier forest landscape | Mammalia |
| 2021 | Umaru Bangura | Lassa Virus Circulation in Small Mammal Populations in Bo District, Sierra Leone | Biology |
