# Supplementary material for: Rodent trapping studies as an overlooked information source for understanding endemic and novel zoonotic spillover
Source: PLoS Negl Trop Dis. 2023 Jan 23;17(1):e0010772. doi: 10.1371/journal.pntd.0010772 (PMC9894545; doi:10.1371/journal.pntd.0010772)
Supplement: S3 Table — (DOCX) [file pntd.0010772.s003.docx]

## Supplementary Table 3

S3A Table: Final GAM model (5). TN_density_ ~ Tweedie(P_density_ + R_area_ + ψ_urban_ + (X * Y)“)

| **Component** | **Term** | **Estimate** | **Std Error** | **t-value** | **p-value** |  |
| --- | --- | --- | --- | --- | --- | --- |
| A. parametric coefficients | (Intercept) | -2.599 | 0.123 | -21.112 | 0.0000 | *** |
| **Component** | **Term** | **edf** | **Ref. df** | **F-value** | **p-value** |  |
| B. smooth terms | s(pop_2005) | 7.131 | 11.000 | 6.398 | 0.0000 | *** |
|  | s(area_km2) | 3.629 | 9.000 | 3.450 | 0.0000 | *** |
|  | s(urban) | 1.921 | 9.000 | 1.235 | 0.0019 | ** |
|  | s(x,y) | 27.252 | 39.000 | 4.563 | 0.0000 | *** |
| Signif. codes: 0 <= '***' < 0.001 < '**' < 0.01 < '*' < 0.05 | | | | | | |
|  | | | | | | |
| Adjusted R-squared: 0.301, Deviance explained 0.487 | | | | | | |
| fREML : 3907.563, Scale est: 8.408, N: 1450 | | | | | | |

S3B Table: GAM model 1. TN_density_ ~ Tweedie(X * Y)

| **Component** | **Term** | **Estimate** | **Std Error** | **t-value** | **p-value** |  |
| --- | --- | --- | --- | --- | --- | --- |
| A. parametric coefficients | (Intercept) | -2.062 | 0.126 | -16.403 | 0.0000 | *** |
| **Component** | **Term** | **edf** | **Ref. df** | **F-value** | **p-value** |  |
| B. smooth terms | s(x,y) | 30.431 | 39.000 | 7.290 | 0.0000 | *** |
| Signif. codes: 0 <= '***' < 0.001 < '**' < 0.01 < '*' < 0.05 | | | | | | |
|  | | | | | | |
| Adjusted R-squared: 0.0261, Deviance explained 0.349 | | | | | | |
| fREML : 4252.745, Scale est: 11.991, N: 1450 | | | | | | |

S3C Table: GAM model 2. TN_density_ ~ Tweedie(P_density_ + (X * Y))

| **Component** | **Term** | **Estimate** | **Std Error** | **t-value** | **p-value** |  |
| --- | --- | --- | --- | --- | --- | --- |
| A. parametric coefficients | (Intercept) | -2.230 | 0.119 | -18.704 | 0.0000 | *** |
| **Component** | **Term** | **edf** | **Ref. df** | **F-value** | **p-value** |  |
| B. smooth terms | s(pop_2005) | 2.818 | 11.000 | 7.231 | 0.0000 | *** |
|  | s(x,y) | 27.640 | 39.000 | 4.545 | 0.0000 | *** |
| Signif. codes: 0 <= '***' < 0.001 < '**' < 0.01 < '*' < 0.05 | | | | | | |
|  | | | | | | |
| Adjusted R-squared: 0.161, Deviance explained 0.422 | | | | | | |
| fREML : 3924.187, Scale est: 9.836, N: 1450 | | | | | | |

S3D Table: GAM model 3. TN_density_ ~ Tweedie(P_density_ + R_area_ + (X * Y))

| **Component** | **Term** | **Estimate** | **Std Error** | **t-value** | **p-value** |  |
| --- | --- | --- | --- | --- | --- | --- |
| A. parametric coefficients | (Intercept) | -2.371 | 0.120 | -19.699 | 0.0000 | *** |
| **Component** | **Term** | **edf** | **Ref. df** | **F-value** | **p-value** |  |
| B. smooth terms | s(pop_2005) | 3.182 | 11.000 | 9.055 | 0.0000 | *** |
|  | s(area_km2) | 3.052 | 9.000 | 2.294 | 0.0000 | *** |
|  | s(x,y) | 26.819 | 39.000 | 4.843 | 0.0000 | *** |
| Signif. codes: 0 <= '***' < 0.001 < '**' < 0.01 < '*' < 0.05 | | | | | | |
|  | | | | | | |
| Adjusted R-squared: 0.148, Deviance explained 0.443 | | | | | | |
| fREML : 3941.346, Scale est: 9.432, N: 1450 | | | | | | |

S3E Table: GAM model 4. TN_density_ ~ Tweedie(P_density_ + ψ_tree_ + ψ_urban_ + (X * Y)

| **Component** | **Term** | **Estimate** | **Std Error** | **t-value** | **p-value** |  |
| --- | --- | --- | --- | --- | --- | --- |
| A. parametric coefficients | (Intercept) | -1.775 | 0.123 | -14.379 | 0.0000 | *** |
| **Component** | **Term** | **edf** | **Ref. df** | **F-value** | **p-value** |  |
| B. smooth terms | s(cropland) | 0.000 | 9.000 | 0.000 | 0.7706 |  |
|  | s(shrubland) | 0.000 | 8.000 | 0.000 | 0.4709 |  |
|  | s(tree_cover) | 1.579 | 9.000 | 0.795 | 0.0089 | ** |
|  | s(urban) | 4.772 | 9.000 | 19.703 | 0.0000 | *** |
|  | s(x,y) | 1.837 | 19.000 | 1.499 | 0.0000 | *** |
| Signif. codes: 0 <= '***' < 0.001 < '**' < 0.01 < '*' < 0.05 | | | | | | |
|  | | | | | | |
| Adjusted R-squared: 0.0599, Deviance explained 0.306 | | | | | | |
| fREML : 4198.018, Scale est: 12.895, N: 1450 | | | | | | |
