# Supplementary material for: Rodent trapping studies as an overlooked information source for understanding endemic and novel zoonotic spillover
Source: PLoS Negl Trop Dis. 2023 Jan 23;17(1):e0010772. doi: 10.1371/journal.pntd.0010772 (PMC9894545; doi:10.1371/journal.pntd.0010772)
Supplement: S1 Fig — Green points represent the start date of rodent trapping studies, blue points representing the final trapping activity. Red points indicate the publication of studies. Increasing numbers of studies have been published since 2000 with more studies being conducted over repeated visits. (DOCX) [file pntd.0010772.s004.docx]

## Supplementary Fig 1


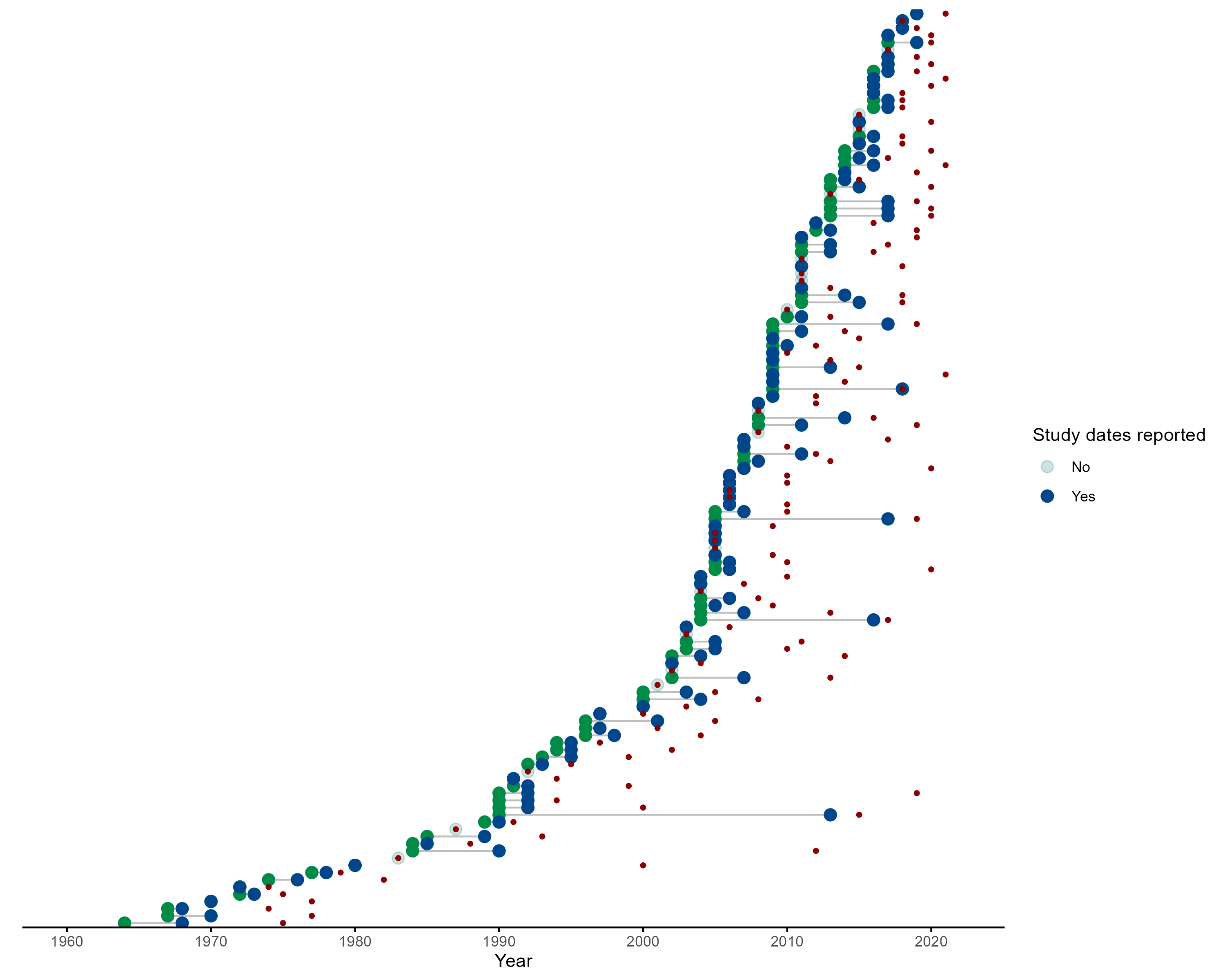


Supplementary Fig 1. Timeline of included studies. Green points represent the start date of rodent trapping studies, blue points representing the final trapping activity. Red points indicate the publication of studies. Increasing numbers of studies have been published since 2000 with more studies being conducted over repeated visits.
