# Supplementary material for: Rodent trapping studies as an overlooked information source for understanding endemic and novel zoonotic spillover
Source: PLoS Negl Trop Dis. 2023 Jan 23;17(1):e0010772. doi: 10.1371/journal.pntd.0010772 (PMC9894545; doi:10.1371/journal.pntd.0010772)
Supplement: S2 Fig — Brown regions represent areas with higher than expected trapping effort, green regions represent areas lower than expected trapping effort. Basemap shapefile obtained from GADM 4.0.4 [38]. (DOCX) [file pntd.0010772.s005.docx]

## Supplementary Fig 2


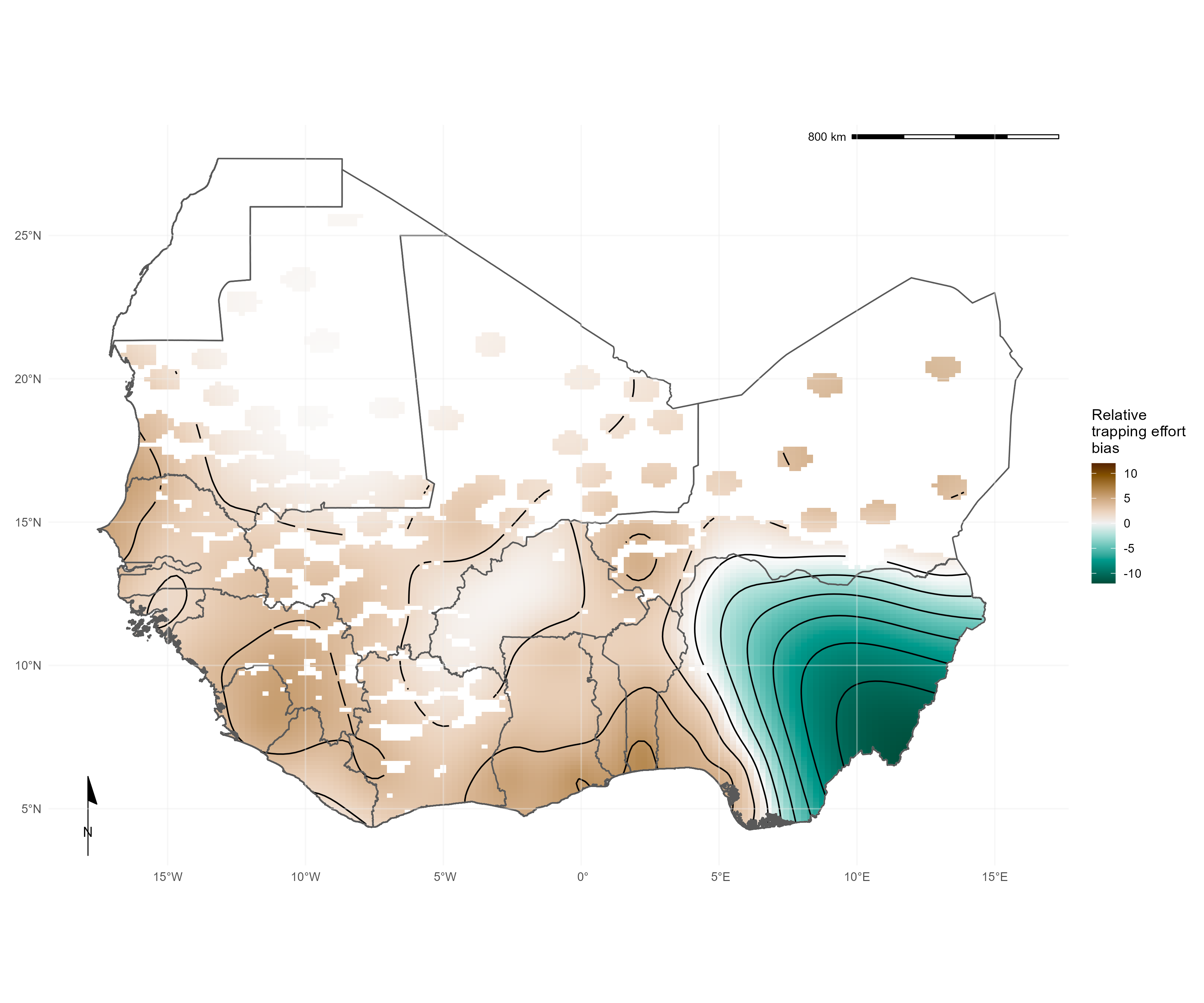


Supplementary Fig 2. Relative trapping effort bias across West Africa from the subset of included studies reporting trapping effort, adjusted for proportion urban land cover and proportion tree cover. Brown regions represent areas with higher than expected trapping effort, green regions represent areas lower than expected trapping effort. Basemap shapefile obtained from GADM 4.0.4 [38].
