# Supplementary material for: Rodent trapping studies as an overlooked information source for understanding endemic and novel zoonotic spillover
Source: PLoS Negl Trop Dis. 2023 Jan 23;17(1):e0010772. doi: 10.1371/journal.pntd.0010772 (PMC9894545; doi:10.1371/journal.pntd.0010772)
Supplement: S4 Fig — A) Identified host-pathogen associations at pathogen family level through detection of acute infection (i.e. PCR, culture). B) Identified host-pathogen associations at pathogen family level through serological assays (i.e. ELISA). Percentages and colour relate to the proportion of all assays that were positive. Associations with a black border are present in the CLOVER dataset. (DOCX) [file pntd.0010772.s007.docx]

## Supplementary Fig 4


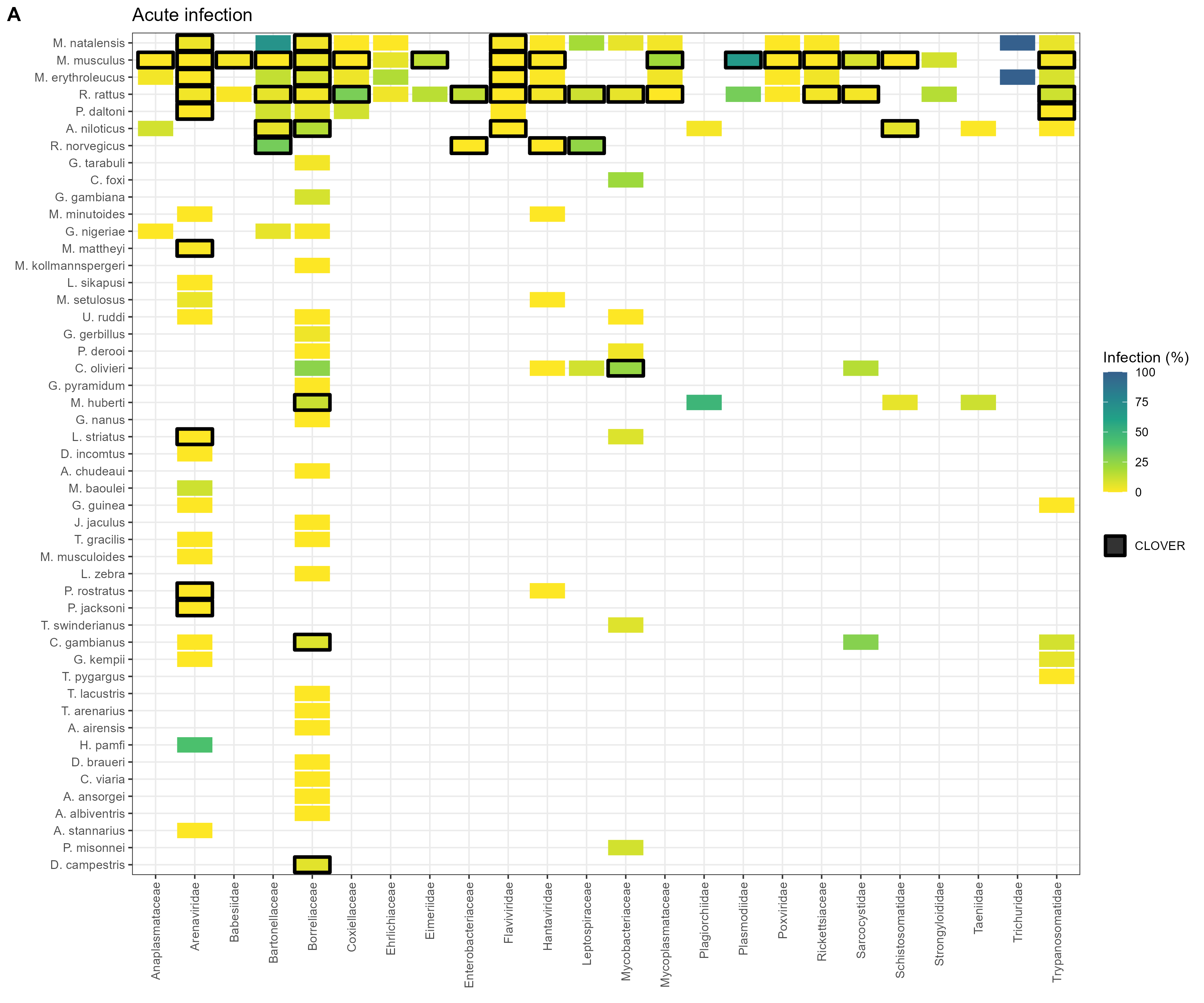


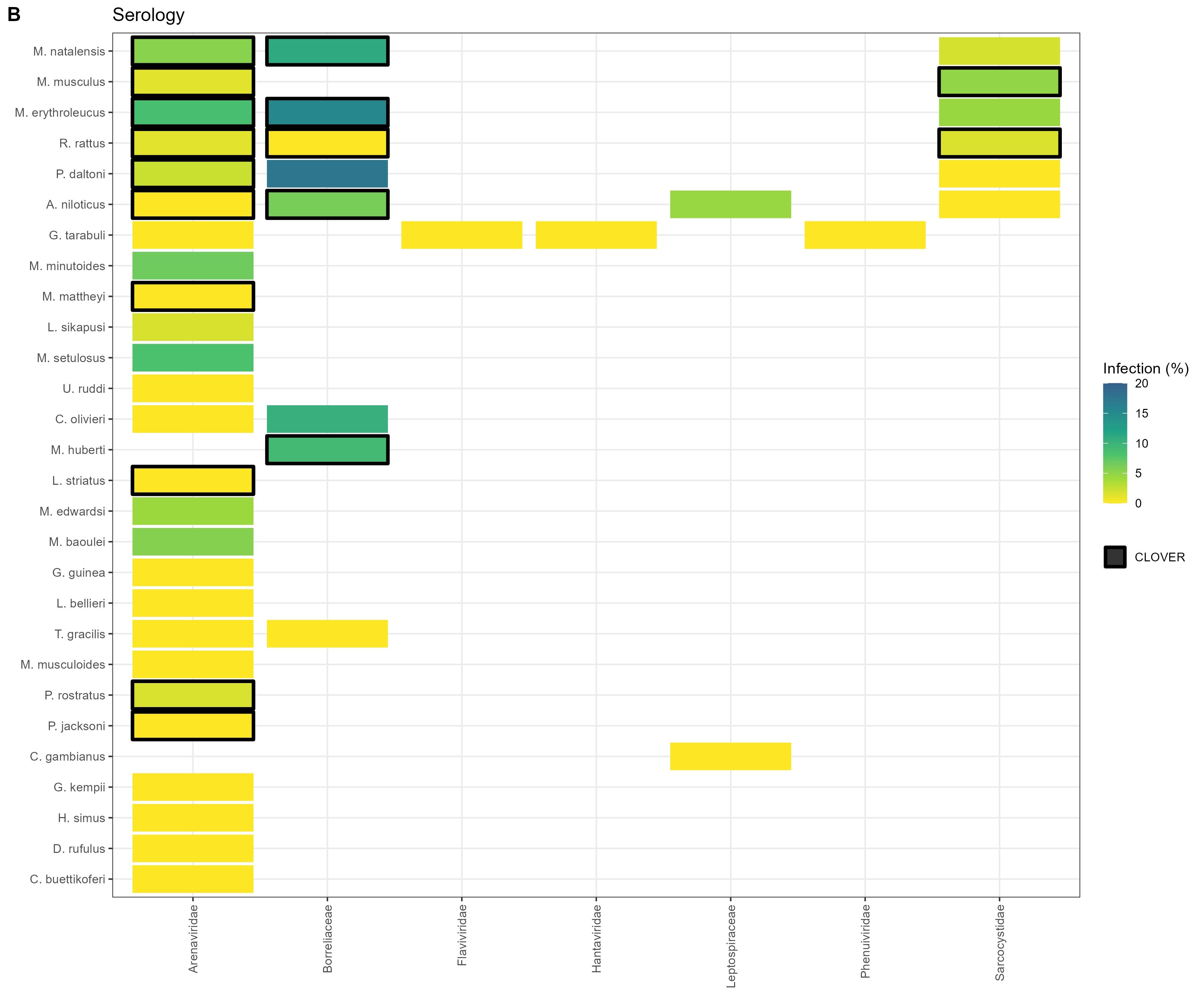


Supplementary Fig 4. A) Identified host-pathogen associations at pathogen family level through detection of acute infection (i.e. PCR, culture). Percentages and colour relate to the proportion of all assays that were positive. Associations with a black border are present in the CLOVER dataset. B) Identified host-pathogen associations at pathogen family level through serological assays (i.e. ELISA). Percentages and colour relate to the proportion of all assays that were positive. Associations with a black border are present in the CLOVER dataset.
