# Supplementary material for: Rodent trapping studies as an overlooked information source for understanding endemic and novel zoonotic spillover
Source: PLoS Negl Trop Dis. 2023 Jan 23;17(1):e0010772. doi: 10.1371/journal.pntd.0010772 (PMC9894545; doi:10.1371/journal.pntd.0010772)
Supplement: S5 Fig — Basemap shapefile obtained from GADM 4.0.4 [38]. (DOCX) [file pntd.0010772.s008.docx]

## Supplementary Fig 5


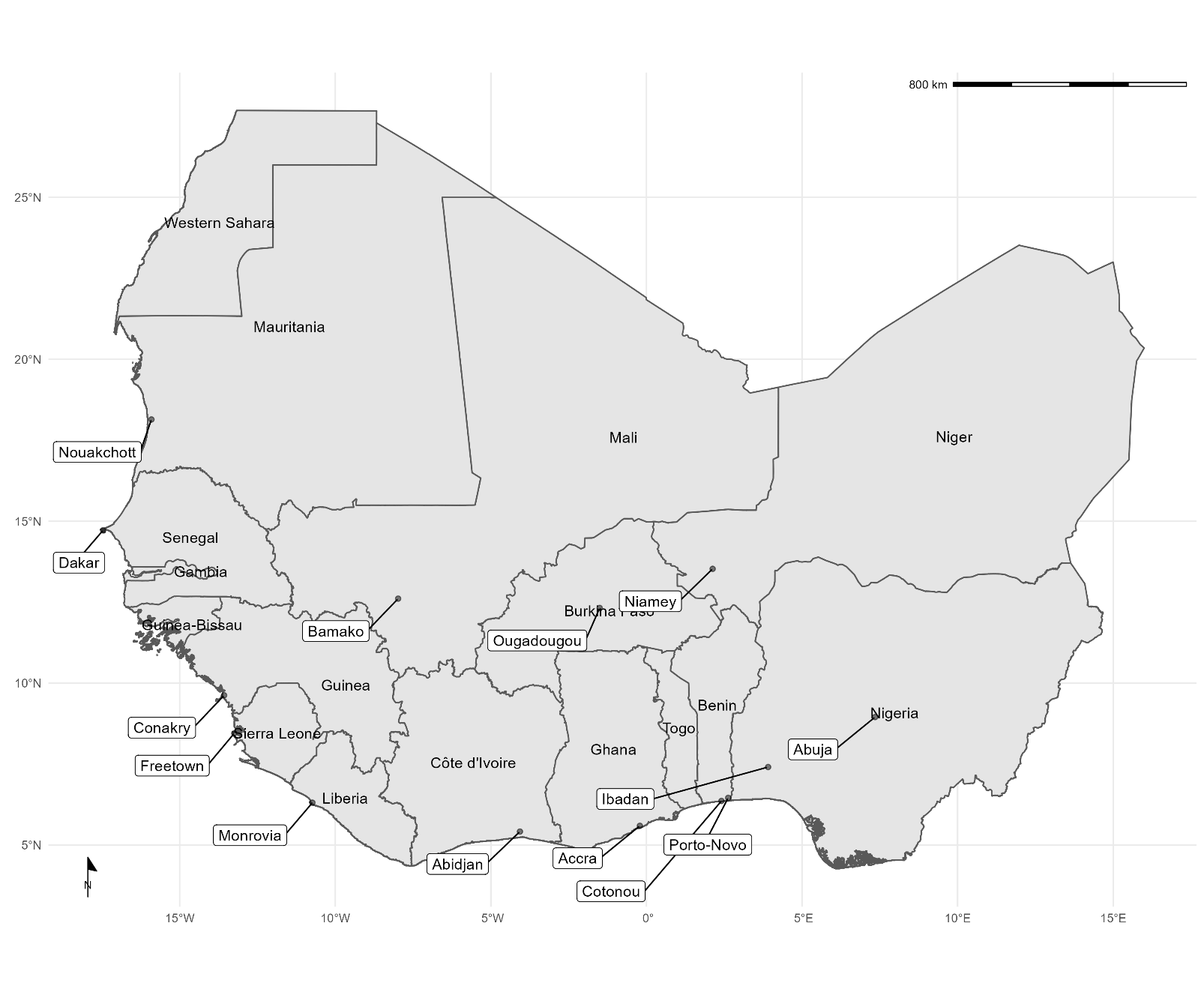


Supplementary Fig 5. A map of the study region with capital cities and areas discussed in the manuscript highlighted. Basemap shapefile obtained from GADM 4.0.4 [38].
