# Supplementary material for: Rodent trapping studies as an overlooked information source for understanding endemic and novel zoonotic spillover
Source: PLoS Negl Trop Dis. 2023 Jan 23;17(1):e0010772. doi: 10.1371/journal.pntd.0010772 (PMC9894545; doi:10.1371/journal.pntd.0010772)
Supplement: S6 Fig — Each row corresponds to a single rodent species. L) Presence recorded in GBIF (black points) overlaid on IUCN species range (red-shaded area). R) Detection (purple) and non-detection (orange) from rodent trapping studies overlaid on IUCN species ranges. M. musculus has no IUCN West African range. Basemap shapefile obtained from GADM 4.0.4 [38]. (DOCX) [file pntd.0010772.s009.docx]

## Supplementary Fig 6


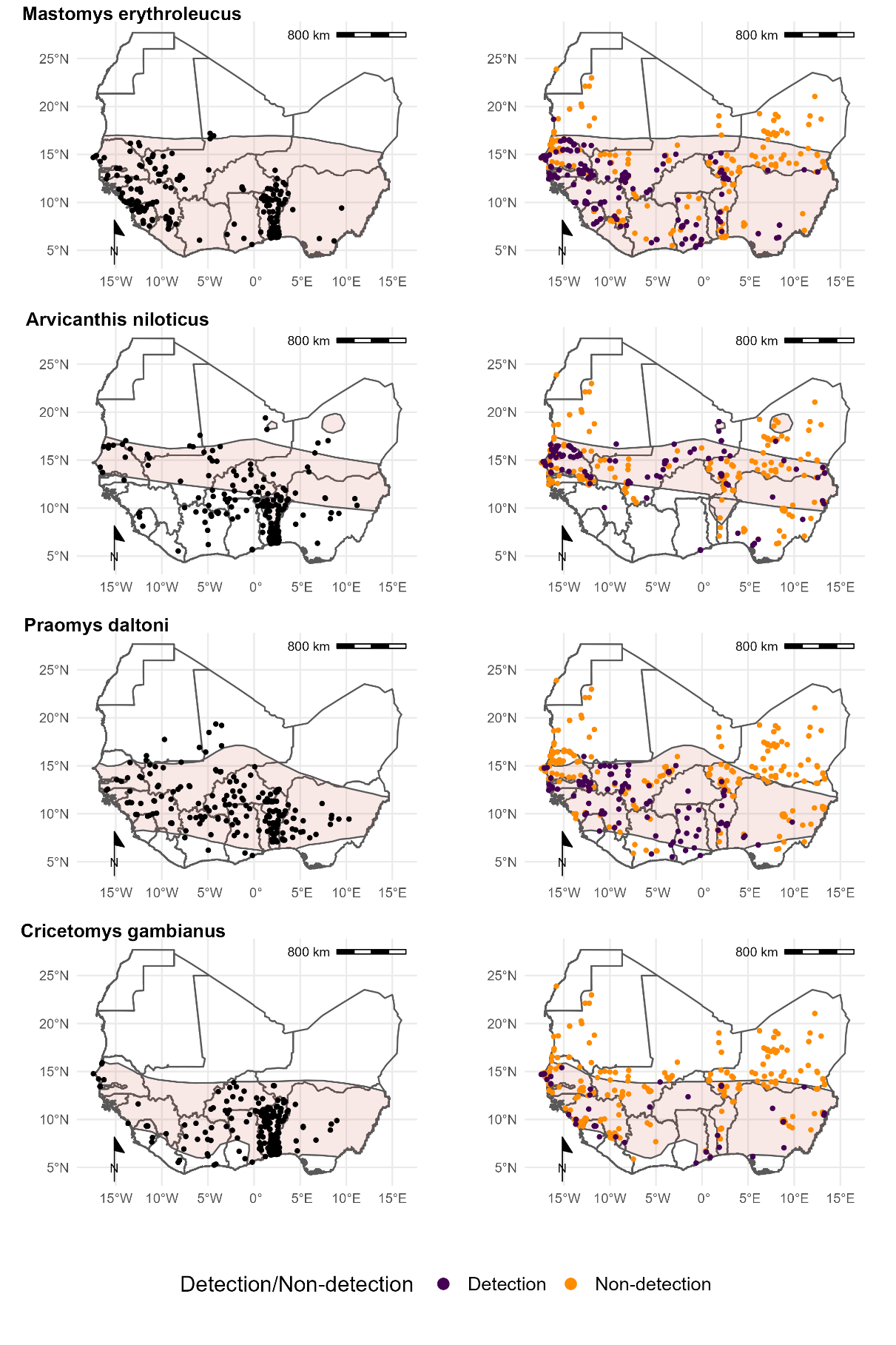


Supplementary Fig 6. Locations of detection and non-detection sites for rodent species in West Africa. Each row corresponds to a single rodent species. L) Presence recorded in GBIF (black points) overlaid on IUCN species range (red-shaded area). R) Detection (purple) and non-detection (orange) from rodent trapping studies overlaid on IUCN species ranges. M. musculus has no IUCN West African range. Basemap shapefile obtained from GADM 4.0.4 [38].
