# Supplementary material for: Rodent trapping studies as an overlooked information source for understanding endemic and novel zoonotic spillover
Source: PLoS Negl Trop Dis. 2023 Jan 23;17(1):e0010772. doi: 10.1371/journal.pntd.0010772 (PMC9894545; doi:10.1371/journal.pntd.0010772)
Supplement: Attachment — Submitted filename: reviewer_comments_v5_rk.docx [file pntd.0010772.s010.docx]

**Reviewer comments:**

**Methods**
**Reviewer #1:**

Nil response required

**Reviewer #2:**

Nil response required

**Reviewer #3:** Methods
*General comment: The methods section is clearly written and provides enough detail to follow the experiment. However, the presented study includes data from trapping studies datingback to the 1960ties. However, it is questionable if the inclusion period of the studies over 60 years is really adding validity to the author’s conclusions– since, and as the authors also state – the human population density and distribution, land use and climate changed drastically during this 6 decades in West Africa, and hence also the composition of the rodent fauna in the area most likely changed. Therefore, it would be worth to reconsider limiting this time span to max three decades to increase significance of the presented work. Additionally, it would be an added value to more prominently highlight the number of studies that include information on actual known rodent species of concern here, namely those important for the zoonotic diseases reported on in this manuscript, and considering limiting the exercise to these studies.*

While the human population density, land-use and climate in West Africa have altered since the 1960s the inclusion of rodent studies from this period remains useful to understand the spatial biases in trapping effort in this region. One purpose of this synthesis is to highlight how relatively sparsely sampled data is being relied upon to inform predictions and estimates of current zoonotic risk based on the distribution of rodents across the region. Studies attempting to do this do not typically limit the time period of contributing rodent data, with data from sources such as GBIF going even further back into the 1800’s. We propose it remains useful to compare this to available data from accessible scientific publications.

The following sentence on line 196 has been added to indicate the number of studies explicitly stating aims to investigate rodent hosts of known or proposed zoonoses.

**“Of these, 55 (43%) were conducted to investigate rodent-borne zoonoses, with the remaining 77 (57%) conducted for ecological purposes (i.e. population dynamics, distribution) in rodents, including those known to be hosts of zoonotic pathogens.”** (Line 196-198)

As above, we propose it is worth retaining studies that do not assess for zoonotic pathogens as they contain important data on rodent occurrence and trapping effort that can be used to improve studies using rodent distributions to assess zoonosis risk.

*Line 164 Here, action is needed by adding clear information on what the authors refer to as “relevant studies”.*

This has been changed to:

**“For included studies with available data we extracted information on all microorganisms and known zoonotic pathogens tested and the method used (e.g., molecular or serological diagnosis).”** (Line 180-181)

*Lines 164-171 From reading this paragraph, it is not clear on what basis and to what extend any “microorganisms and zoonotic pathogens” were included, and how “all microorganisms tested” are relevant for a manuscript reporting on zoonotic disease spill over. The authors are encouraged to revise to allow for more clarity and easier understanding for the reader.*

While the manuscript focusses on the use of rodent data for the investigation of potential zoonotic pathogen spillover, many studies investigating the risk of pathogen emergence rely on rodent host distributions to inform their risk models. Perhaps this has not been set up adequately in the introduction of the work and so the following has been added to the introduction:

The section on the impact of sampling bias on inference has been expanded. “These sampling biases can importantly distort produced species distribution **models that are used to infer risk of zoonotic disease risk** [20]. Datasets on host-pathogen associations also can suffer from biases introduced from literature selection criteria and taxonomic discrepancies **resulting in differential likelihood of accurate host-pathogen attribution by host species.**” (Line 93-97)

The reason for including “all microorganisms” rather than limiting to known zoonotic pathogens is due to several included studies only identifying microorganisms to Family classification (i.e. Arenaviridae or Borreliaceae) which may include known zoonotic pathogens (i.e. *Lassa mammarenavirus* or *Borrelia crocidurae*) or may be detection of non-pathogenic members of these families. For the primary analysis of host-pathogen associations in section 5.3 only known pathogens were included. This limitation was relaxed in the supplementary material (Supplementary figure 4a and 4b) as it is possible that detected microorganisms included pathogen species. For the primary analysis of host testing for potential pathogens (section 5.4) these higher order families were included to highlight the limited proportion of a rodent species expected range where these micro-organisms and known pathogen have been investigated. To clarify this distinction the following sentence was added to the methods section:

A section on the terms of zoonosis and micro-organisms as used in this manuscript was added. **“A broad definition of known zoonotic pathogen was used, a species of micro-organism carried by an animal that may spread to humans and cause illness [CDC].** **We do not include evolved pathogens acquired originally through zoonotic pathways in our definition (i.e. HIV). The term microorganism is used where either the microorganism is not identified to species level, in which case it remains unclear whether it is a zoonotic pathogen (i.e. Arenaviridae), or the species is not known to be a zoonotic pathogen (i.e. *Candidatus Ehrlichia senegalensis*).”** (Line 183-188)

Changes have been made in several places to limit reference to pathogen only when talking about known zoonotic pathogen species. In places where reference to multiple species, some of which may be non-pathogenic the term microorganism is preferred.

*Line 172-182 This paragraph reports on the data sources as identified through the review process and includes also certain analysis and results – therefore consider a separate heading for this paragraph.*

A new section 4.1.2. with the heading **Description of included studies*.*** Has been added to clearly indicate this is analysis of the combined data.

**Results**
**Reviewer #1:**

Nil response required

**Reviewer #2:**

Nil response required

**Reviewer #3:**

*Figure 3 The presentation of Mus musculus data rises several questions here. There seems to be a very limited overlap of “detection” data between the GBIF information and the trapping studies. GBIF presents occurrence data covering Benin, while the trapping studies could not detect any Mus musculus in Benin. Can the authors explain this discrepancy?*

The occurrence of *Mus musculus* in Benin has been entered into GBIF by Armand Kingbo at the Laboratory of Forest Sciences in Benin based on a document entitle Biodiversity Atlas of West Africa (2014). Benin. Tome 1. Volume 1. with the following GBIF DOI <https://doi.org/10.15468/h7rqo9>. Data in our study for Benin comes from 7 studies which trapped solely in Benin and 3 studies that trapped in multiple countries. Only 2 of the Benin based studies reported on *Mus musculus* presence or absence. The remaining 5 studies did not detect *Mus musculus* at any of their trapping sites but did not explicitly report its absence and so based on our methods do not contribute a non-detection point (as it is not explicitly stated that they detected none of that species). The three multi-country studies that did not detect *Mus musculus* in Benin did report *Mus musculus* presence elsewhere in their studies and so the absence of a reported individual of this species is interpreted as a non-detection. It is not possible to infer why the results from the data contributing to GBIF is vastly different from that observed from trapping studies although highlighting these discrepancies is important, for example based on a GBIF dataset the risk to human populations from a zoonotic pathogen for which *Mus musculus* is a host will be vastly different if a dataset based on rodent trapping studies is used.

2 studies did report *Mus musculus* individuals trapped in the port area of Cotonou, unfortunately, this is not currently visible in Figure 3. due to the order of plotting points**.** Figure 3. has been modified so detection will always be shown on top of non-detection.

*Table 2 The headings of the columns need attention: please add clear information on what the numbers report (for example the column “Tested” shall include “Tested (N)” etc.)*

These changes have been made.

**Conclusions**
**Reviewer #1:**

Nil response required

**Reviewer #2:**

*Assume that these coverage values are based on the proportion of raster cells to which a point trapping location was allocated, in which case the values are highly dependent on the raster size chosen, which is a limitation that should be made clear.*

We have highlighted the effect of the raster cell size choice in the discussion and agree that while the values are highly dependent on this it is unlikely to change the finding of sparse sampling of all rodent species.

**“Our estimates of the proportion of a rodent species range that have been sampled along with pathogen testing within their sampled range are sensitive to our choice of raster cell size, smaller area cells will reduce the reported coverage while larger cells will have the opposite effect. Despite this, the observed patterns are unlikely to importantly change, with the finding of sparse sampling of both rodents and their pathogens remaining present across cell scales.”** (Line 453-458)

*One of the aims is to establish sampling bias in relation to human population density and land use. However, it is not made clear in the conclusions why the predicted trapping effort is of more use use than simply identifying geographic areas with less sampling (e.g. benefits of Figure 2 rather than identifying gaps in Figure 1). E.g. It may be useful to increase sampling in non-urban areas with lower human density, but this is not clearly discussed.*

This has been expanded on in the discussion with the advantages of the modelling based approach highlighted.

**“In addition to identifying point locations of prior rodent and pathogen sampling (Figure 1.), additional information on the trapping effort (density of trap-nights), human population density and land use type have been incorporated to produce a value of relative effort that will assist researchers in identifying specific locations where predictions based on these underlying data sources may suffer from increased effects of trapping effort bias. This approach improves the ease of identifying under sampled locations, for example, Figure 1. may suggest that South East Senegal, Southern Mali and Southern Niger are well sampled based on locations of trapping sites. When the number of trap nights, human population density and land use of these regions are taken into account (Figure 2.) and compared with better sampled locations (i.e. Western Senegal, Eastern Sierra Leone) these areas are found to be relatively under sampled and would benefit from further sampling effort. This contrasts to North West Nigeria where no trapping has occurred (Figure 1.), the modelling approach has perhaps highlighted this beyond a simple map of currently trapped locations as an immediate priority for sampling of rodents and their pathogens given high human population densities and a human dominated landscape.”** (Line 435-449)

**Reviewer #3:**

*The discussion is a great reading and brings to the point a variety of challenges authors faced while performing this nicely designed study.
However, I encourage the authors to revise it in view of my general comments to the manuscript.*

*Lines 386-388 In addition to the number of rodents trapped please also provide the information of how many species these belonged to.*

Changed as suggested.

Here we have synthesised data from 126 rodent trapping studies containing information on more than 72,000 rodents, **from at least 132 species of small mammals**, across 1,611 trap sites producing an estimated 942,669 trap nights across 14 West African countries. (Line 418-421)

**Editorial and Data Presentation Modifications?**
**Reviewer #1:**

*In the figure 1A and B, country and city names mentioned in the main text should be indicated for readers who are not familiar with West African countries and their cities.*

Adding this information to Figure 1A. obscured the data. A new supplementary figure (Supplementary Figure 5.) was produced which labels the countries and highlights the locations of capital cities and cities mentioned in the text.

**Reviewer #2:**
Nil response required

**Reviewer #3:**

Nil response required

**Summary and General Comments**
**Reviewer #1:**

*The present manuscript submitted by D Simons et al. to the PLOS Neglected Tropical Diseases journal addressed to review and summarize the previously published trapping studies targeting rodents and pathogens they carry in Western African countries. Since the individual studies may not be able to discover anything beyond their study focus and area, I really love to read this kind of studies challenging to extract the gaps of them. The study seems to be firmly performed and presentation and interpretation of data are fair.

A major issue from a virological standing point is that the overlapping between a pathogen group (Arenaviridae family) and a pathogen (Lassa mammareanavirus). Since other three targets in the present manuscript (Borreliaceae, Leptospiraceae, and Toxoplasma gondii) are all independent and not overlapped each other, the incongruity of selection of pathogens and pathogen groups may not be acceptable, especially by virologists. The authors may want to find which arenavirus was detected in the studies detecting Arenaviridae family and divide them into Lassa mammarenavirus and the others.*

This is an important point, unfortunately the included studies that reported Arenaviridae or Arenavirus sp. did so as the microorganisms detected were not identified down to species level. Potentially a large number of these were in fact *Lassa mammarenavirus* but it is not possible to classify them as such based on data reported by the study authors so it would be prudent to maintain the classification that authors reported. As these records contribute ~3,000 tested rodents and show a similar pattern of limited testing within rodent ranges it would be a shame to remove them entirely despite the overlapping nature of including Family level arenavirus and species level arenavirus infections.

The following sentence was added to section 5.4 to clarify this **“Studies that reported Arenaviridae infection did not identify the microorganism to species level and were distinct from those reporting *Lassa mammarenavirus* infection”** (Line 399-401)

*“wildlife defaunation” may not be suitable to be listed here since this should be a result of “intensifying anthropogenic pressure”.*

This has been changed to “**leading to altered rodent species assemblages.”** To focus on the potential species and individual abundance changes that may occur under the previous factors (Line 66-67)

**Reviewer #2:**

*This is a well-written and useful synthesis of rodent trapping studies in West Africa, that has identified new host-pathogen associations and potential gaps in our understanding of host and pathogen distributions, with clear public health relevance.*

*The introduction could benefit from clarifying the difference in how curated data sets (e.g. GBIF and CLOVER) obtain records and the considered trapping studies. E.g. Are these trapping studies not previously included in curated databases because of data access issues?*

A sentence has been added to describe how GBIF and IUCN data is typically incorporated “**These data are typically obtained from museum specimen collections and non-governmental organisation surveys.**” (Line 91-93) The generation of CLOVER is described in more detail in the referenced manuscript and section 4.2.3. in the methods section, with mention of the literature extraction process in the introduction (Line 95).

*Line 70- Is 4 days the correct number here? This is too short for a gestation period*

This is likely an error, although the source states 4 days. This will be kept as short gestation with no number of days mentioned.

*Line 227- May be useful to explain the use of Tweedie here.*

The following sentence was added “**Spatial aggregation of relative trapping effort was described using an exponential dispersion distribution (Tweedie).**” (Line 248-249) with a reference 41[<https://doi.org/10.1016/S0304-3800(01)00494-X>].

**Reviewer #3:**

*While the study works on relevant questions, the way it is reported in this manuscript is not suitable for publication. The way we scientists report about zoonotic diseases and the animals affected needs to be carefully revised. As in this manuscript, the authors report about “rodents” in an indiscriminate way – a very diverse and ecologically very important group of animal species, that play an important role in the ecosystem. While only a few rodent species are known to play a role in zoonotic disease transmission, the reaction and actions taken by people based on such generalized ways of communicating information will and are mostly targeting any rodent species, with a huge negative impact on their abundance, diversity and occurrence. However, rodents are much more than “…important globally distributed reservoirs of known and novel zoonotic pathogens”. I am a very strong advocate for change in our human – animal relationship, and here, I see that we scientists have an important role to play, including in the way we communicate. The rodent’s population dynamic is heavily impacted on by habitat changes and landscape modulations caused by humans and this is equally applying for their health, fitness and exposure to pathogens. To achieve health for all – what in my reading includes human, animal, plant and environmental health, we scientist have an important role to play and be sensitive in the way we communicate. Therefore, I encourage the authors to carefully revise their manuscript with a One Health lens to avoid any inappropriate generalization of “rodents” and rather focus on promoting a better understanding of human activities and its impact on animals and zoonotic pathogens.*

We agree with these comments and have attempted to frame this article through a “One Health” lens focussing on anthropogenic factors altering rodent species assemblages which may result in species that host zoonotic pathogens being more abundant and widespread. In response to this not coming across as clearly as I would have like I have reworded a couple of sections.

**Abstract**

“Rodents **a diverse, globally distributed and ecologically important order of mammals are nevertheless** important reservoirs of known and novel zoonotic pathogens. Ongoing anthropogenic land use change is altering the composition **of these species’ abundance and distribution which among zoonotic host species may alter** the risk of zoonoses spillover events. These changes **demand** **that a better** understanding of the current distribution of rodent species is vital for **mitigating potential zoonotic disease hazard and managing risk**.” (Line 15-21)

**“**Here, we synthesise data from West Africa from 127 rodent trapping studies, published between 1964-2022, as an additional source of information to characterise the range and presence of **rodent species and identify the subgroup of species that are potential or known pathogen hosts**” Line (25-26)

**Introduction**

“Rodents, **an ecologically vital order of mammals** carry a disproportionate number of zoonotic pathogens and are abundant across West Africa.”

“**Most rodent species do not provide a direct risk to human health** and all species provide important and beneficial ecosystem services including pest regulation and seed dispersal [12]. **Increasing risks of zoonotic spillover events are driven by human actions rather than by rodents, for example, invasive rodent species being introduced to novel ranges through human transport routes.** Rodents typically demonstrate “fast” life histories **which allow them to exploit opportunities provided by anthropogenic disturbance** [13].” Line (73-79)

“Currently within West Africa, **some** **rodent species are known to be** **involved** in the transmission of multiple endemic zoonoses with large burdens on human health, these pathogens include Lassa fever, Schistosomiasis, Leptospirosis and Toxoplasmosis [26,27]. **The presence of other species within shared habitats may mitigate the spread of these pathogens through the “dilution effect”, ongoing loss of biodiversity may further increase the risk to human populations** [5].” Line (107-113)

The assessment of risk from rodent borne zoonoses and methods to mitigate this are beyond the scope of this manuscript. Here, we have attempted to quantify the spatial biases and data issues in available datasets that underly other work that does attempt to discuss this. We believe that improving the quality of the science behind assessment of zoonotic risk will allow a better understanding of the complex interplay between rodent species (including host and non-host species) and human health.

The choice of “rodents” rather than focussing on specific species is primarily because the sampling has been biased towards several species and so the lack of testing and evidence from other species does not allow for clear cut distinction between host- and non-host species. It is my preference that we continue to use this higher-level term throughout because of this.
