# Supplementary material for: Rodent trapping studies as an overlooked information source for understanding endemic and novel zoonotic spillover
Source: PLoS Negl Trop Dis. 2023 Jan 23;17(1):e0010772. doi: 10.1371/journal.pntd.0010772 (PMC9894545; doi:10.1371/journal.pntd.0010772)
Supplement: Attachment — Submitted filename: reviewer_comments_minor_changes.docx [file pntd.0010772.s011.docx]

Lines 413 - 416:

In response to the point raised during revision round 1, the authors now state that the more than 72k trapped rodents belong to “at least 132 species of small mammals”. The IUCN defines the group of small mammals to comprise rodents, tree shrews and eulipotyphlans – the latter two therewith being non-target species groups for the presented study.

Here, the authors shall provide clarity if the study included information of non-rodent species as well (including three shrews and eulipotyphlans), or otherwise clearly state how many of the “at least 132 species” were actually rodents and how many non-rodent small mammals.

*Changed as suggested*

Since the authors did not modify the figures 1A and B as I suggested to add names of countries and cities "in the figures", I could not judge whether the addition will obscure the data or not. The authors should show the figures which were actually obscured. Addition of the new supplementary figure 5 did not improve the readability than google maps.

*Figure 1A has been updated to show country names.*

Line 16- needs comma after rodents

Line 18- Composition of these species' abundance does not make sense. Suggest removing "the composition"

Line 20- "demand that a better understanding of the current distribution of rodent species is vital". Suggest rewording.

*Changed as suggested*
